# Adding Genetic Testing to Evidence-Based Guidelines to Determine the Safest and Most Effective Chronic Pain Treatment for Injured Workers

**Published:** 2015-12

**Authors:** Brian Meshkin, Katrina Lewis, Svetlana Kantorovich, Natasha Anand, Lisa Davila

**Affiliations:** 1 Proove Biosciences, Inc., Irvine, CA, USA;; 2 Spokane Spine Center, Spokane WA, USA

**Keywords:** chronic pain, opioids, genetic testing, substance abuse, injured workers

## Abstract

Published guidelines for treating injured workers include the need for personalized treatment to manage chronic pain symptoms and increase functional status. However, they often fail to clarify how to objectively personalize these treatments. Further, certain patients need analgesic relief beyond the short term. In these cases, it is not sufficient or reasonable to utilize the typical broad protocol-based justifications for reduction of opioids and other medications in a haphazard manner based purely on poor response, without attempting to elucidate possible pharmacogenetic reasons for this. These guidelines acknowledge the problem of substance abuse and set forth methods for treatment and prevention. Although it has been established in the scientific community that an individual’s experience of pain and likelihood for addiction both have genetic components, genetic testing is not routinely included as part of the overall treatment plan for injured workers with chronic pain. Because decisions in cases of workplace injury should be based on scientific evidence, genetic testing results can add some objective information to the existing subjective and objective clinical data; help ascertain the efficacy and potential for toxicity of treatment; and therefore provide more information for accurate clinical decisions. We propose the addition of genetic testing to consensus guidelines for treating injured workers in order to improve patients’ functional status, increase productivity, improve safety of prescribing, decrease the likelihood of substance abuse, and save on overall healthcare costs.

## INTRODUCTION

Guidelines currently used in decision making for workplace injuries include the Guidelines for the Chronic Use of Opioids by the American College of Occupational and Environmental Medicine (ACOEM), the Official Disability Guidelines (ODG), and the Medical Treatment Utilization Schedule (MTUS) for the State of California.

An expert multidisciplinary panel used evidence-based data to develop the ACOEM guidelines to manage injured workers whose pain has not been controlled by more conservative means. The ODG is a product of the Work Loss Data Institute (WLDI), an independent database development company focused on workplace health and productivity, and provides evidence-based disability duration guidelines and benchmarking data for reportable conditions. The MTUS is an adaptation of the ODG for the State of California. All of the guidelines contain detailed information about treating injured workers, including claimants who have long-term disability related to chronic non-cancer pain (CNCP) and opioid use.

In this paper, we propose that such guidelines could be enhanced by integrating genetic testing into treatment plans for these claimants. We will describe challenges facing CNCP claimants and clinicians and describe how genetic information may guide treatment decisions and thus result in better outcomes for injured workers with CNCP.

### Workplace Injuries: Scope and Associated Sequelae

National surveillance information indicates that there are about 3 million workers injured each year (1). The U.S. Bureau of Labor Statistics (BLS) reports that overall incidence of nonfatal occupational injury and illness cases requiring days away from work was 109.4 cases per 10,000 full-time workers in 2013 (2). Research shows, however, that many workplace-related injuries are not reported by employees or employers, and some are not captured by BLS or state-level workers compensation reporting bodies (3). Thus the resultant disability and time away from work may have a more profound effect on individual health; lost productivity; and indirect and direct costs. 

### Chronic Pain from Workplace Injuries

About one-third of work-related injuries necessitating days away from work are related to musculoskeletal conditions (2). Pain related to these injuries can be self-limited (acute-resolving when the injury resolves), subacute pain (usually defined as lasting 4 to 12 weeks) or become chronic pain, which persists for over 4 months or years. Among workers compensation (WC) claimants who have back pain, one of the most common CNCP conditions, up to 20% are still collecting benefits after at least one month following the date of injury (4).

### Opioids for pain: benefit or detriment?

While opioids may have a pain-relieving benefit in the short-term for some musculoskeletal injuries, this class of medication is not specified in guidelines as a necessary treatment in the acute post-injury period (5). Further, opioids have not been shown to have a significant effect on CNCP (6, 7); nor have these drugs been associated with overall improved physical or emotional functioning. A recent systematic review showed that opioid therapy for CNCP patients has been associated with specific complications including fractures, myocardial infarction, sexual dysfunction, opioid abuse, and opioid overdose (8). WC claimants who take opioids have been shown to have prolonged disability and higher overall costs than claimants who were never prescribed these drugs (9, 10). This is the case especially in the case of long-acting opioids, such as OxyContin^®^ (11-14).

### Genetics, pain, and addiction

Research evidence indicates that along with external factors, genetics have a significant effect on an individual’s pain perception (15, 16). In addition, the National Institute on Drug Abuse (17) and the American Society of Addiction Medicine (18) report that along with addiction-related environmental factors such as stress, sexual abuse, and drug availability, the likelihood for addiction is about 50% related to an individual’s genetic profile. Genetic variations including single nucleotide polymorphisms (SNPs) that affect neurochemistry in the mesocorticolimbic or “reward” pathway, have been implicated for their link to opioid reward, craving, and addiction (19-23).

Despite growing knowledge in this area, genetic testing is not currently incorporated into the standard of care for injured workers described in the three sets of guidelines we reviewed. We believe that this neglect of genetic factors has in part contributed to opioid addiction; prolonged disability; delayed return-to-work rates among workers compensation claimants; and increased employer and payer costs.

### Personalized Medicine

Over the past decade, there has been a concerted attempt to recognize the convergence of genetic factors and disease processes and to coordinate care accordingly. This concept is commonly referred to as personalized medicine (24), an approach that may be useful for CNCP management—especially in instances of opioid use. Within the field of personalized medicine, pharmacogenomics or pharmacogenetics refers to the practice of using results of genetic testing to guide medication management decisions (21, 25-31).

### Genetic testing to establish level of pain

The use of genetic testing is mentioned in the consensus guidelines only with regard to diagnosing pain. The guidelines are clear that cytokine DNA testing should not be used to diagnose pain. MTUS guidelines, for example, are very explicit: “There is no current evidence to support the use of cytokine DNA testing for the diagnosis of pain, including chronic pain (32)”. There are, however, non-cytokine-based genetic tests based on the analysis of SNPs, such as catecholamine-O-methyltransferase (COMT), which can predict the likelihood of individual pain sensitivity (15, 16, 33-35).

### Genetic testing to screen for likelihood of opioid abuse

Approximately 30% of WC claimants are taking opioids (36), putting a significant number of America’s workers at risk for addiction. Consensus guidelines explicitly recommend screening for addiction risk before to initiating opioid therapy (32). It is important to identify individuals who have the potential to develop aberrant drug use both prior to prescribing opioids and while actively undergoing this treatment. Such screening tends to occur after a claimant is already taking opioids on a chronic basis, and consists of screens for aberrant behavior/misuse (32) rather than intrinsic addiction risk.

In the consensus guidelines, experts have established a validation threshold for substance abuse risk screening. Five screening methods are listed as options: The CAGE Questionnaire (37); Skinner Trauma Screen (38); Cyr-Wartman Screen (39); Screener and Opioid Assessment for Patients in Pain, Revised (SOAPP-R) (40); and the Opioid Risk Tool (ORT) (41). There are approximately 14 additional tools available. Studies show, however, that even when physicians use such screening tools, their ability to predict patients who misuse prescription opioid pain medications is no better than 50% (42, 43).

### Genetic Testing to Guide Long-term Opioid Use

For long-term or chronic use of opioids-defined as longer than 6 months-several consensus guidelines provide instructive insights. The ACOEM guidelines indicate that screening for addiction risk should occur, but if opioids are providing pain relief and improved functioning, they should be continued, even if a patient is at risk for addiction (44). In this case, if genetic testing confirms an increased addiction risk, the patient may require closer monitoring.

On the topic of acute or sub-acute pain ODG suggests that longer duration of use may lead to mental dependency; higher prevalence of work disability; depression; anxiety; and substance abuse (45). In such instances, genetics may need to be evaluated because results may objectively show a predisposition to addiction, lack of responsiveness to certain opioids, or potential for early tolerance. Again, the treatment plan may likely change.

For chronic pain, the ODG suggests opioids only as a second- or third-line option at doses less than or equal to 120 milligrams daily oral morphine equivalent dosage in patients not at risk of misuse or diversion. Genetic studies suggest, however, that patients with certain gene variants may require less morphine equivalent doses to achieve the same analgesic response as wild type patients, obviously dependent on which opioid is utilized (46). In addition, drug tolerance is associated in part with how medications interact and are metabolized (47) and genetic testing may provide information about the activity of enzymes involved in drug metabolism (48). This includes the role of other non-analgesic medication metabolites and competing pathways in individual patient polypharmacy to delineate possible objective reasons for poor patient response to specific medication regimes and drugs. This might provide further impetus for rationalization by providers of medication regimens, and thereby improve on both safety and costs.

## METHODS

### Framework for Adding Genetic Testing to Inform Decisions Prior to Opioid Therapy Consideration

We propose a framework that integrates genetic testing in the following way (Table 1):

**Table 1:** Potential Role of Genetic Testing before Opioid Therapy

Steps Based on Consensus Guidelines	Should genetic testing be a part of the step?

1. Attempt to determine if the pain is nociceptive or neuropathic, and if there are underlying contributing psychological issues.	Yes
2. Attempt non-opioid analgesic trial prior to opioids.	Yes
3. Set goals and establish opioid contract.	Yes
4. Baseline pain and functional assessments should be made (including psychological).	Yes
5. Establish likelihood of opioid weaning in the event of treatment failure.	Yes
6. Use periodic urine drug screens.	Yes
7. Use a consistent pharmacy.	No
8. Discuss the risks and benefits of the use of controlled substances with the patient, caregiver, or guardian.	Yes

**1. Attempt to determine if the pain is nociceptive or neuropathic and if there are underlying contributing psychological issues **(32). Despite imaging studies and other diagnostics, CNCP can appear to have no discernible cause (5) thus differentiating between the two types of pain may be problematic. Nociceptive pain is a response to injury and can arise from somatic or visceral pain receptors. Opioids tend to be effective in relieving this type of pain. Neuropathic pain, on the other hand, signals damage to nerves but nevertheless opioids tend to be ineffective. Consequently, if patients with neuropathic pain take opioids for an extended period of time, they can respond poorly or develop hyperalgesia (49).

Patients with neuropathic pain, however, have been shown to experience improved pain relief with selective serotonin reuptake inhibitors (SSRIs) and anticonvulsants in some instances (50). This may be due in part to pain pathways that differ from those of nociceptive pain. Research using rat models shows that neuropathic pain results in changes in the serotonin and dopamine systems, which may also be an early sign of chronic maladaptation to ongoing pain (51).

Genetic tests can reveal information regarding specific SNPs in these serotonin and dopamine pain pathways. Hence clinicians have objective data to aid in determining whether a patient is prone to a particular etiology of pain, and their metabolism and response to certain anticonvulsants and SSRIs, which may help to avoid trial-and-error treatments (52, 53).

Along with determining the type of pain, a comprehensive psychological assessment may reveal the presence of disorders that are commonly associated with CNCP including depression, anxiety, and bipolar disease (54-58). Treating co-morbidities can improve pain perception, lower pain responses, and thereby improve psychological functioning (59), but in some instances the clinician may not be aware that a patient has a co-occurring psychiatric disorder. Genetic testing can show if a patient has likelihood for one or more comorbidities. Numerous studies have found significant associations between specific SNPs and psychiatric conditions including substance abuse (Table 2).

**Table 2:** Genetic Variations and Associated Neuro-Psychiatric Disorders

Gene	Disorder	

5-Hydroxytryptamine (Serotonin)) Receptor 2A, G protein-coupled	Alcohol abuse (78)	• Neuropathic pain (82)
	Anxiety (79)	
	Depression (80)	
	Drug Abuse (81)	
Solute Carrier Family 6 (Neurotransmitter Trans-porter), Member 3	Alcohol abuse (83)	• Substance abuse (84)
Catechol-O-Methyl Transferase	Alcohol abuse (85)	• Methamphetamine abuse (87)
	Major depression (86)	• Schizophrenia (88)
Dopamine D2 Receptor	Alcohol, cocaine, nicotine dependence (89)	
Dopamine D1 Receptor	Depression (90)	• Heroin addiction (91, 92)
Dopamine D4 Receptor	Anxiety (93, 94)	• Drug abuse (95)
Solute Carrier Family 6 (neurotransmitter trans-porter	Cocaine addiction (96), Methamphetamine addiction (97)	
Dopamine Beta Hydroxylase	Alcohol abuse (43)	• Schizophrenia (99, 100)
	Cocaine addiction (43, 98)	• Smoking addiction (101)
Methylene Tetrahydrofolate Reductase	Bipolar disorder, depression, schizophrenia (102)	
Human Kappa Opioid Receptor	Alcohol abuse (103)	• Mood disorders (104)
Gamma Aminoburyric Acid A Receptor, gamma 2	Alcohol abuse (105)	• Methamphetamine dependence (106)
Opioid Receptor, Mu 1	Complex regional pain syndrome (107)	• Heroin dependence (108)

**2. Attempt non-opioid analgesic trial prior to opioids** (32). Genetic testing may influence the choice of non-opioid analgesics. For example, testing for SNPs that affect cytochrome P450 enzymes may influence response or toxicity to nonsteroidal anti-inflammatory medications (NSAIDS), which are commonly prescribed as a first-line treatment for pain (60). To screen for risk of NSAID-related gastrointestinal bleeding, physicians could determine the presence of a cytochrome P450, family 2, subfamily C, polypeptide 9 genotype that has been associated with this complication (61). Again, more careful selection of patients at risk for GI bleeding with NSAIDs could help avoid this potentially catastrophic and expensive complication.

**3. Set goals for opioid therapy and establish a written pain agreement to improve documentation of patient education, the treatment plan, and informed consent** (32). Prescription drug monitoring programs and other initiatives that help detect drug abuse can be useful for detecting medication-related problems after they have occurred, but prevention, of course, is ideal (62). Patient education can be enhanced by discussions about how an individual’s personal genetic profile may provide insights into the risk of opioid abuse and how genes affect drug metabolism. This, along with the addition of genetic testing to informed consent forms, could make such pain agreements comprehensive and more meaningful to patients.

**4. Perform baseline pain and functional assessments** (32). Assessment of function should include social; physical; and psychological factors; and also daily and work activities, and should be performed using a validated instrument or numerical rating scale (32). As clinical recovery may be dependent upon identifying and addressing previously unknown or undocumented medical and/or psychosocial issues (63), genetic testing for relevant SNPs may provide insights into undiagnosed conditions that directly affect physical or emotional functioning, including heightened pain sensitivity and the quality of a patient’s coping mechanisms (64). This could help guide decisions in regards to referral of certain patients for more intensive cognitive behavioral therapy, which has a strong evidence base for efficacy in CNCP (65). A patient’s risk of opioid abuse should be also be included as part of the genetic assessment, as numerous studies support the link between mesolimbic SNPs and opioid abuse (66).

**5. Assess the likelihood that the patient could be weaned from opioids if there is no improvement in pain and function** (32). Because genetics may correlate with opioid dependence, they are likely to contribute to a patient’s ability to be weaned from such medication (67). Weaning or discontinuing opioid therapy without a functional restoration or alternative maintenance regimen (either buprenorphine or methadone) for CNCP patients has been characterized by high dropout rates (68) and can be hindered by the presence of diagnosed or undiagnosed psychiatric conditions (69), inadequate emotional support (70) or the inability to cope with withdrawal symptoms (71). There is also a lack of formal evidence-based opioid-weaning clinical guidelines for clinicians’ use (72). Thus, having information about SNPs that may affect withdrawal severity, or indicate the likelihood of maintenance therapy success may help clinicians develop a plan that will help patients maintain physical and emotional comfort and develop effective coping mechanisms while tapering off opioids.

**6. Consider the use of a urine drug screen to assess for the use or the presence of illegal drugs.** Genetic variations in drug metabolizing enzymes can influence the metabolism of medications, thus affecting the accuracy of urine drug screening results (73). Therefore, it may be appropriate to consider this form of genetic testing to explain idiosyncratic results (74).

**7. Use a consistent pharmacy (75).**

**8. The physician should discuss the risks and benefits of controlled substances and other treatment modalities with the patient, caregiver, or guardian** (32). Discussing the risks and benefits of opioid therapy with the patient may lessen the chances of abuse or diversion of the prescribed opioid (62). In addition, there have been legal precedents established with regard to genetic or hereditary conditions that support inclusion of genetic testing in such discussions and/or written agreements (76, 77). Courts have determined that physicians must disclose all risks to patients, and ignorance does not excuse responsibility.

## GENETIC TESTING CHALLENGES

Genetic testing to guide chronic pain treatment is not yet routine and more prospective outcome studies on the value of this practice are needed. Further, some experts believe that genetic information is only moderately predictive of the likelihood of addiction and that such information is being used prematurely before it has been shown to be a valid part of a treatment plan. However, emerging data shows that genetic prediction of addiction risk maybe far superior to the conventional validated risk tools mentioned above and currently in use. Nevertheless, we believe pharmacogenomics has potential value for individualizing patient treatment. Genetic testing, as part of the course of treatment, can give clinicians objective data for assessment of disease risk, diagnosis, and medication response—all of which could lead to better pain relief and improved functioning for CNCP patients.

## CONCLUSION

With the emerging consensus that genetic predisposition is involved in pain perception and about half of substance abuse cases, it appears plausible that genetic testing could play an important role in guiding treatment decisions in chronic pain therapies for injured workers, especially if opioid therapy is already underway or being considered. Evaluating a patient’s genetic profile may give clinicians objective information on which to base treatments, thereby decreasing pain; improving functioning; improving safety by reducing drug interactions, complications, and toxicities; helping return claimants to work; and saving costs for employers and payers.
